# Dense calcification in a GH-secreting pituitary macroadenoma

**DOI:** 10.1530/EDM-13-0079

**Published:** 2014-02-01

**Authors:** Ramez Ibrahim, Atul Kalhan, Alistair Lammie, Christine Kotonya, Ravindra Nannapanenni, Aled Rees

**Affiliations:** Royal Hallamshire HospitalSheffieldUK; 1 University Hospital of WalesCardiffUK; 2 Cardiff UniversityCardiffUK; 3 Bronglais HospitalAberystwythUK; 4 Institute of Molecular and Experimental Medicine, Cardiff UniversityCardiffUK

## Abstract

A 30-year-old female presented with a history of secondary amenorrhoea, acromegalic features and progressive visual deterioration. She had elevated serum IGF1 levels and unsuppressed GH levels after an oral glucose tolerance test. Magnetic resonance imaging revealed a heterogeneously enhancing space-occupying lesion with atypical extensive calcification within the sellar and suprasellar areas. Owing to the extent of calcification, the tumour was a surgical challenge. Postoperatively, there was clinical, radiological and biochemical evidence of residual disease, which required treatment with a somatostatin analogue and radiotherapy. Mutational analysis of the aryl hydrocarbon receptor-interacting protein (*AIP*) gene was negative. This case confirms the relatively rare occurrence of calcification within a pituitary macroadenoma and its associated management problems. The presentation, biochemical, radiological and pathological findings are discussed in the context of the relevant literature.

**Learning points:**

- Calcification of pituitary tumours is relatively rare.
- Recognising calcification in pituitary adenomas on preoperative imaging is important in surgical decision-making.
- Gross total resection can be difficult to achieve in the presence of extensive calcification and dictates further management and follow-up to achieve disease control.

## Background

Calcification is an uncommon feature of pituitary adenomas and extensive calcification evident radiologically is especially rare. Between 0.2 and 14% of pituitary adenomas display radiological evidence of calcification (1) (2), but microscopic calcification is more common, occurring in 5.4–25% of the cases (3) (4). Eleven cases have been described in the literature, comprising five patients with prolactinomas (5) (6) (7) (8) (9), three patients with growth hormone (GH)-secreting adenomas (4) (10) (11), two patients with TSHomas (12) and one patient with a gonadotrophin-secreting tumour (13). It is important to recognise the presence of calcification in pituitary adenomas, as it may influence the choice of surgical approach (transsphenoidal vs transcranial). Furthermore, complete resection may be difficult, leading to the need for other treatments to control disease activity.

## Case presentation

A 30-year-old female presented with a history of secondary amenorrhoea and gradual onset of visual deterioration over a period of 4 months. On review, she was found to have prominent acromegalic features including ‘spade-like’ hands, prognathism, increased inter-dental spacing, skin tags, nasal enlargement, and pigmentation around the eyes, neck and flexures. She also reported excessive sweating and weight gain despite exercise and gave a past history of carpal tunnel syndrome. There was no personal or family history of prior endocrine disease.

## Investigation

**Figure 1:**
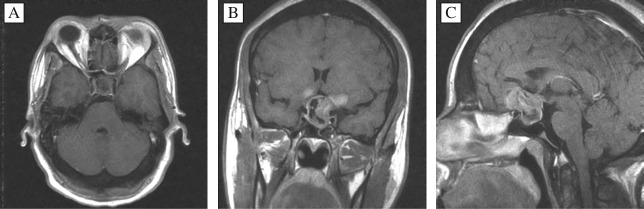
Preoperative T1-weighted axial (A), coronal (B) and sagittal (C) gadolinium-enhanced MRI scan.

Formal visual field assessment revealed a bitemporal visual field loss. Biochemical testing revealed markedly raised insulin-like growth factor 1 (IGF1) levels (208 ng/ml; reference range 15.3–43.1 ng/ml), suppressed gonadotrophin levels, marginally raised prolactin (PRL) levels (569 mU/l; reference range 50–560 mU/l) and normal thyroid function (thyroid-stimulating hormone (TSH) levels 1.33 mU/l and free thyroxine levels 14.9 pmol/l). Serum calcium levels were normal (2.37 mmol/l). Cortisol response to 250 μg of Synacthen was normal (baseline, 196 nmol/l and 30 min, 673 nmol/l). A glucose tolerance test confirmed failure to suppress serum GH levels (nadir, 60.6 μg/l). Magnetic resonance imaging (MRI) of the pituitary revealed a pituitary macroadenoma with sellar and suprasellar extension (Fig. 1). The tumour was of grade IV-D according to the modified Hardy classification.

**Figure 2:**
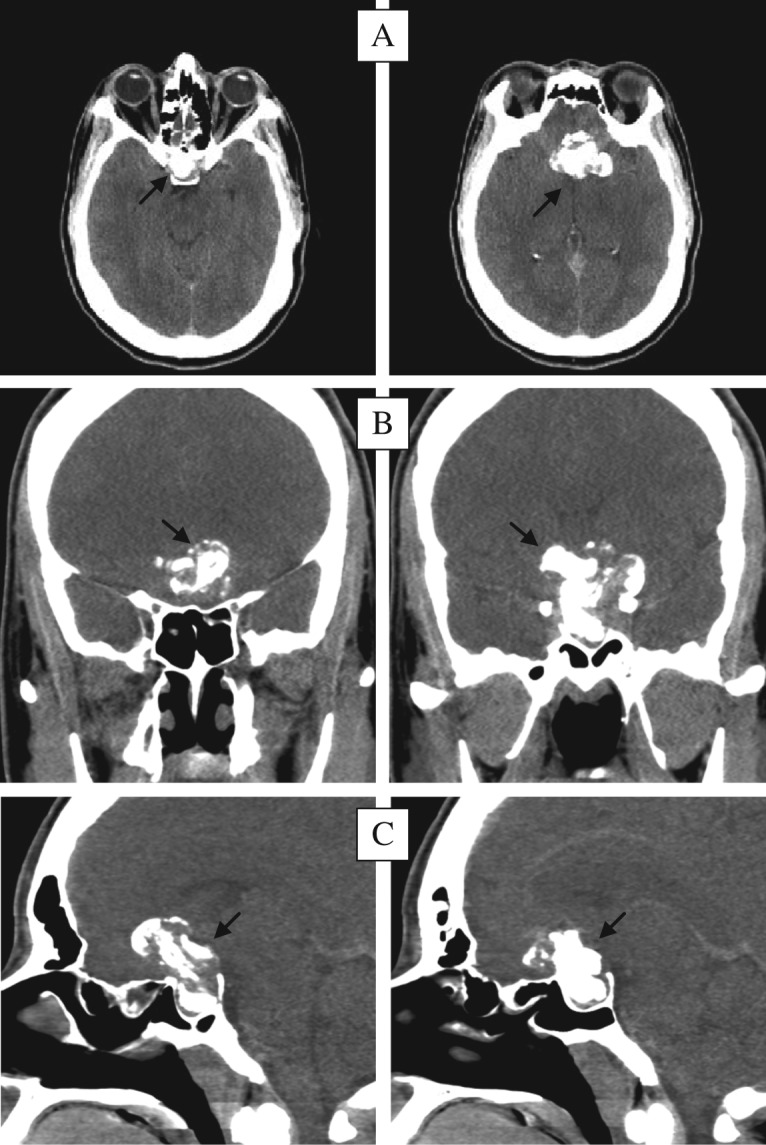
Axial (A), coronal (B) and sagittal (C) CT head images showing the pituitary adenoma with suprasellar and parasellar extension and extensive calcification (black arrows).

Preoperative computed tomography (CT) of the head confirmed a large, heavily calcified tumour in the pituitary fossa reaching the chiasmatic cistern. The tumour extended underneath the inferior medial aspects of both frontal lobes and anteriorly above the planum sphenoidale (Fig. 2).

## Treatment

The clinical, biochemical and radiological findings were consistent with a GH-secreting pituitary macroadenoma causing mild stalk compression hyperprolactinaemia. Somatostatin analogue therapy (octreotide LAR 10 mg monthly i.m. injections) was commenced, and the patient was scheduled for an elective craniotomy, with a view to decompressing the optic nerves and chiasm and to reduce the tumour bulk.

**Figure 3:**
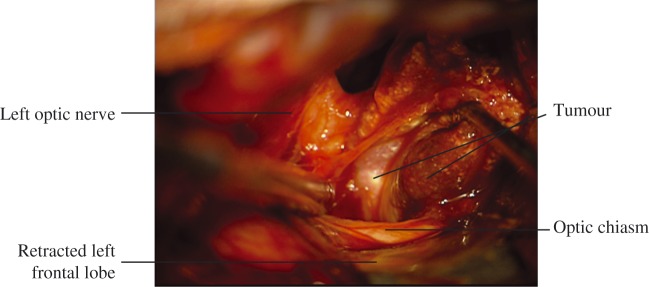
Intraoperative image showing a dense calcified tumour dissected off the compressed left optic nerve and chiasm.

**Figure 4:**
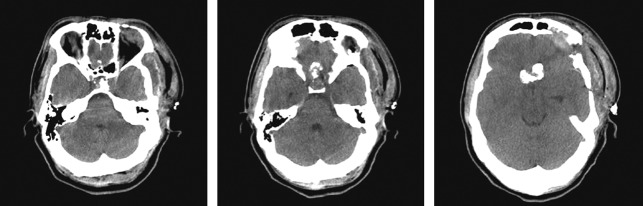
Immediate postoperative period axial CT head images showing the extent of surgical resection with residual calcified tumour.

A left fronto-temporal craniotomy and a sub-frontal approach were followed. During surgery, the tumour was found to have a granular gritty texture with ‘stone-hard’ areas and interspersed soft tumour components. Decompression of the optic nerves/chiasm was achieved, with removal of the majority of the suprasellar tumour component and dissection of a densely calcified part off the left optic nerve (Fig. 3). Complete removal was not possible due to the adherence of densely calcified tumour to the left sellar/cavernous region and inferior medial frontal lobes (Fig. 4).

**Figure 5:**
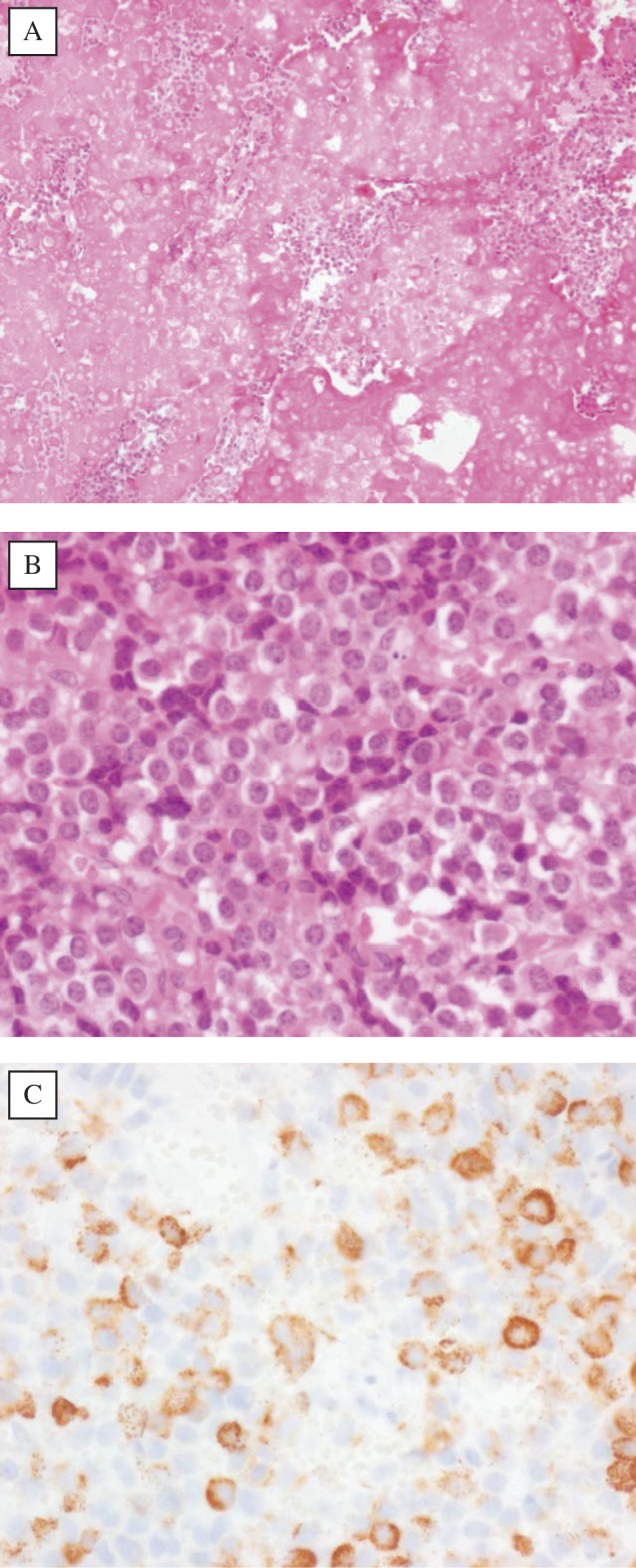
(A) Extensive confluent calcification with small islands of intervening tumour cells (haematoxylin and eosin (H&E) staining; ×100); (B) high-power view of tumour cells, which have mildly pleomorphic nuclei with no significant mitotic activity or necrosis (H&E staining; ×400) and (C) patchy tumour cell GH immunoreactivity (GH antibody staining; ×400).

Histology revealed heavily calcified tissue with intervening sheets of mildly pleomorphic pituitary adenoma (Fig. 5A) and reticulin network effacement but no necrosis or significant mitotic activity (Fig. 5B). Immunohistochemical staining revealed widespread GH staining (Fig. 5C) and only minor patchy PRL staining. The tumour did not stain for follicle-stimulating hormone, luteinizing hormone (LH), adrenocorticotrophin or TSH. The Ki67 proliferation index was <5%, and p53 antibody staining was negative. The overall features were consistent with a heavily calcified GH-secreting pituitary adenoma.

## Outcome and follow-up

Postoperatively, the patient developed transient diabetes insipidus, which was treated with desmopressin (DDAVP). Ophthalmological examination 1 week after surgery revealed a marked improvement of visual acuity and visual fields. Biochemical testing revealed normal PRL levels and improved but non-normalised IGF1 levels (119 ng/ml) and nadir GH levels following oral glucose administration (7.17 μg/l). She remained euthyroid and euadrenal.

**Figure 6:**
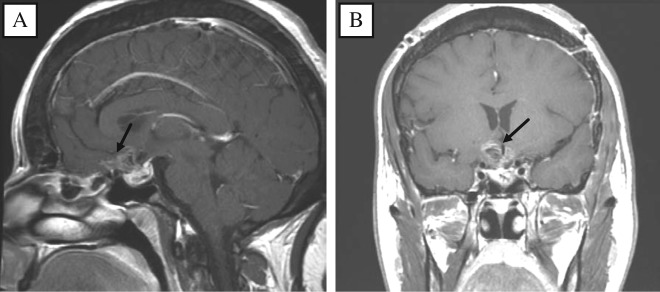
Sagittal (A) and coronal (B) T1 gadolinium-enhanced MRI postoperative images showing enhancing residual tumour (black arrows).

Follow-up gadolinium-enhanced MRI of the head 12 weeks after surgery confirmed a reduction in the size of the pituitary adenoma, but an enhancing residuum was visible within the sella and overlying the planum sphenoidale (Fig. 6).

In view of the residual disease activity and no prospect of surgical cure by re-operation, the patient was recommenced on octreotide LAR therapy, titrated to a maximum dose of 30 mg monthly. After 1 year of therapy, her IGF1 levels had improved only marginally compared with the postoperative values (88 vs 119 ng/ml) and random GH value was 6.02 μg/l. Furthermore, there was no reduction in tumour volume, and hence we elected to proceed to radiotherapy, given as conventional external beam radiotherapy in 28 fractions. Given the patient's young age at presentation and resistance to somatostatin analogue therapy, genetic analysis was carried out for mutations in the aryl hydrocarbon receptor-interacting protein (*AIP*) gene, associated with familial isolated pituitary adenomas, but this was negative. After a further 12 months of follow-up, serum IGF1 levels had returned to normal.

## Discussion

Intracranial calcification is most commonly confined to the basal ganglia, pineal gland, falx, choroid plexus and cerebellum. Such ‘physiological’ calcification occurs to a limited extent, rarely attains a diameter of more than 1 cm, and has a characteristic punctate appearance. Pathological conditions associated with intracranial calcification include trauma, surgery, irradiation, hyper/hypoparathyroidism and tumours. The differential diagnosis of calcified lesions in the sellar region includes craniopharyngioma, meningioma, aneurysm, optic/hypothalamic glioma, germ cell tumours and rarely Rathke's cleft cyst (14) (15). Calcification of pituitary adenoma is relatively rare, with a reported prevalence varying between 0.2 and 14% (1) (2).

The radiological appearance of calcification in pituitary adenomas has been found to be either an intratumoural ‘pituitary stone’ (4) (5) (7) (12) or capsular ‘egg-shell like’ (6) (13). A curvilinear pattern has been suggested to be a feature associated with pituitary adenomas, in contrast to the nodular pattern more commonly observed in craniopharyngiomas (16). The dense and extensive calcification in this case is unusual, as it was both intratumoural and capsular.

Calcification in pituitary adenomas is postulated to be a result of dystrophic calcification due to progressive tumour enlargement with central ischaemic effects, which may also promote osteoid metaplasia when well-differentiated lamellar bone tissue is identified histologically (12) (13). Small foci of calcification can be detected on histological examination in 10–15% of the cases, appearing microscopically as psammoma bodies (2) (9) (17). It has also been suggested that calcification may be induced by secondary degenerative changes as a consequence of silent pituitary apoplexy (8) (13). There may be intraoperative evidence of old bleeding suggestive of prior silent apoplexy (1) (4).

Another possible explanation is induction of heterotopic calcification secondary to tumour compression and increased intrasellar pressure (7) (10). The autocrine and paracrine effects of PRL, GH and molecules such as vascular endothelial growth factor and osteocalcin may also promote calcification (7).

It is important to recognise the presence of calcification within a pituitary tumour as its extent may influence the choice of surgical approach. In previously reported cases, surgery was carried out via a transsphenoidal route, but these cases had a lesser extent of calcification, smaller tumour size, and limited lateral or suprasellar extension. In the present case, the tumour extended to the suprasellar region, with anterior burrowing into both medial frontal lobes above the planum sphenoidale. The extent of calcification, appreciated better on preoperative CT imaging, demanded a craniotomy be performed. The approach was carried out via a left fronto-temporal craniotomy and a sub-frontal approach, because most of the tumour load was on the left.

MRI was not able to verify the true extent of calcification due to the signal dropout of calcium. It should be emphasised that old areas of haemosiderin, which may represent pituitary apoplexy, are also hypointense on T1- and T2-weighted MRI sequences (2). No areas suggestive of recent haemorrhage were encountered during surgery or later observed during histopathological examination.

It appears that calcification can occur in any type of pituitary hormone-producing adenoma. Intense hormonal activity may correlate with the development of intratumoural calcification (5). Most reported cases are PRL-producing adenomas due to their relatively higher frequency (2) (17). The present case confirms the possible occurrence of dense calcification in functioning pituitary adenomas and highlights the importance of preoperative imaging in evaluating such lesions before surgical resection. Gross total resection can be technically challenging in heavily calcified large adenomas. In such cases, medical therapy and/or radiotherapy are usually required to achieve biochemical remission.

## Patient consent

Informed consent was obtained from the patient for publication of this article and accompanying images.

## Author contribution statement

R Ibrahim, A Kalhan and A Lammie contributed to the writing of the initial manuscript. C Kotonya, R Nannapanneni and A Rees revised the final manuscript before submission.
